# Classification, categorization and essential items for digital ulcer evaluation in systemic sclerosis: a DeSScipher/European Scleroderma Trials and Research group (EUSTAR) survey

**DOI:** 10.1186/s13075-019-1822-1

**Published:** 2019-01-24

**Authors:** J. Blagojevic, S. Bellando-Randone, G. Abignano, J. Avouac, L. Cometi, L. Czirják, C. P. Denton, O. Distler, M. Frerix, S. Guiducci, D. Huscher, V. K. Jaeger, V. Lóránd, B. Maurer, S. Nihtyanova, G. Riemekasten, E. Siegert, I. H. Tarner, S. Vettori, U. A. Walker, Y. Allanore, U. Müller-Ladner, F. Del Galdo, M. Matucci-Cerinic, EUSTAR co-workers

**Affiliations:** 1 Department of Experimental and Clinical Medicine, University of Florence, and Department of Geriatric Medicine, Division of Rheumatology and Scleroderma Unit AOUC, Villa Monna Tessa, viale Pieraccini 18, 50139 Florence, Italy; 2 NIHR Leeds Biomedical Research Centre, Leeds Teaching Hospitals NHS Trust and Leeds Institute of Rheumatic and Musculoskeletal Medicine, University of Leeds, Leeds, UK; 3 Rheumatology Institute of Lucania (IReL), Rheumatology Department of Lucania, San Carlo Hospital of Potenza and Madonna delle Grazie Hospital of Matera, Potenza, Italy; 4 Department of Rheumatology, University of Paris Descartes, Paris, France; 5 Department of Rheumatology and Immunology, University of Pécs, Pécs, Hungary; 6 Department of Rheumatology, University College London, Royal Free Hospital, London, UK; 7 Department of Rheumatology, University Hospital Zurich, Zurich, Switzerland; 8 Department of Rheumatology and Clinical Immunology, Kerckhoff-Klinik GmbH, Campus of the Justus-Liebig University Giessen, Bad Nauheim, Germany; 9 Institute of Biometry and Clinical Epidemiology, Charité – Universitätsmedizin Berlin, Corporate member of Freie Universitaet Berlin, Humboldt-Universitaet zu Berlin, and Berlin Institute of Health, Berlin, Germany; 10 Department of Rheumatology, University of Basel, Basel, Switzerland; 11 Clinic of Rheumatology and Clinical Immunology, University of Lübeck, Lübeck, Germany; 12 Department of Rheumatology and Clinical Immunology, Charité – Universitaetsmedizin Berlin, Corporate member of Freie Universitaet Berlin, Humboldt-Universitaet zu Berlin, and Berlin Institute of Health, Berlin, Germany; 13 Rheumatology Section, Department of Precision Medicine, University of Campania “Luigi Vanvitelli”, Naples, Italy

**Keywords:** Systemic sclerosis, Digital ulcers, Essential items, Classification, Categorisation

## Abstract

**Background:**

A consensus on digital ulcer (DU) definition in systemic sclerosis (SSc) has been recently reached (Suliman et al., J Scleroderma Relat Disord 2:115-20, 2017), while for their evaluation, classification and categorisation, it is still missing. The aims of this study were to identify a set of essential items for digital ulcer (DU) evaluation, to assess if the existing DU classification was useful and feasible in clinical practice and to investigate if the new categorisation was preferred to the simple distinction of DU in recurrent and not recurrent**,** in patients with systemic sclerosis (SSc).

**Methods:**

DeSScipher is the largest European multicentre study on SSc. It consists of five observational trials (OTs), and one of them, OT1, is focused on DU management. The DeSScipher OT1 items on DU that reached ≥ 60% of completion rate were administered to EUSTAR (European Scleroderma Trials and Research group) centres via online survey. Questions about feasibility and usefulness of the existing DU classification (DU due to digital pitting scars, to loss of tissue, derived from calcinosis and gangrene) and newly proposed categorisation (episodic, recurrent and chronic) were also asked.

**Results:**

A total of 84/148 (56.8%) EUSTAR centres completed the questionnaire. DeSScipher items scored by ≥ 70% of the participants as essential and feasible for DU evaluation were the number of DU defined as a loss of tissue (level of agreement 92%), recurrent DU (84%) and number of new DU (74%). For 65% of the centres, the proposed classification of DU was considered useful and feasible in clinical practice. Moreover, 80% of the centres preferred the categorisation of DU in episodic, recurrent and chronic to simple distinction in recurrent/not recurrent DU.

**Conclusions:**

For clinical practice, EUSTAR centres identified only three essential items for DU evaluation and considered the proposed classification and categorisation as useful and feasible. The set of items needs to be validated while further implementation of DU classification and categorisation is warranted.

**Trial registration:**

Observational trial on DU (OT1) is one of the five trials of the DeSScipher project (ClinicalTrials.gov; OT1 Identifier: NCT01836263, posted on April 19, 2013).

**Electronic supplementary material:**

The online version of this article (10.1186/s13075-019-1822-1) contains supplementary material, which is available to authorized users.

## Background

In systemic sclerosis (SSc), the pathophysiology is characterised by immune, endothelial and fibroblast dysfunction [1] and microvascular involvement is one of the most important features of the disease [2]. The evolution of vessel involvement frequently leads to tissue ischemia and formation of digital ulcers (DU) that are considered as a significant clinical burden [3, 4] reducing patients’ quality of life [5]. In SSc, the compelling need for a precise definition [6] has eventually led to a consensus on DU definition [7], while for their evaluation, classification and categorisation, an overall agreement is still missing [8].

**Fig. 1:**
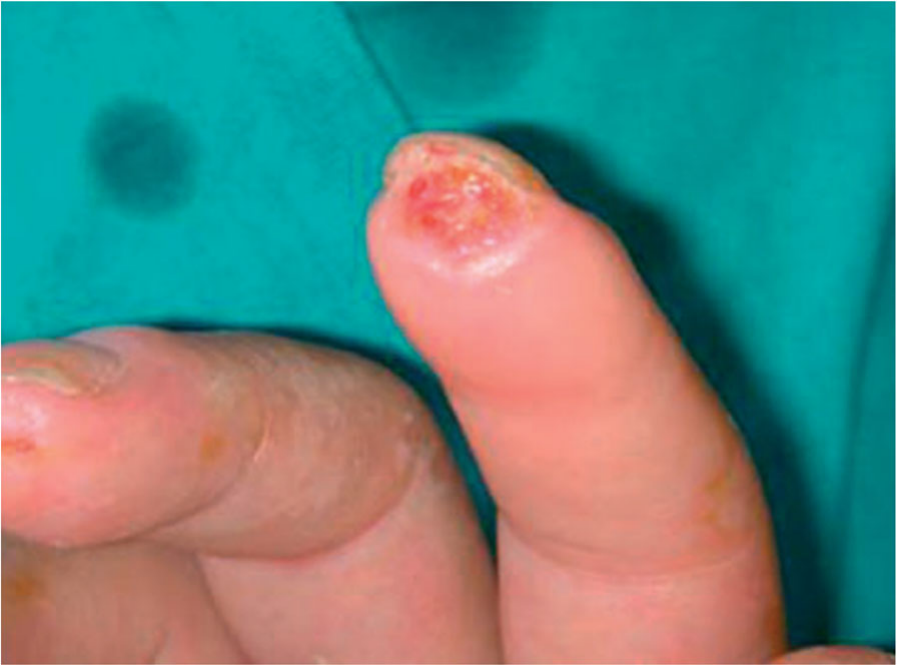
Pure DU due to loss of tissue not dependent from digital pitting scar or calcinosis

Since different types of DU may occur in SSc, a DU classification according to their main features into DU associated digital pitting scars, DU associated with calcinosis, DU due to loss of tissue not associated with DPS or calcinosis (Pure DU) (Fig. 1) and DU associated with gangrene has been proposed [9]. Recently, a new categorisation of DU into episodic, recurrent and chronic DU, derived from the analysis of more than 1400 patients in Europe, has been suggested [3].

DeSScipher is the largest European multicentre project aimed to decipher the optimal management of SSc. It consists of five observational trials (OTs) focusing on DU (OT1), hand arthritis, interstitial lung disease, pulmonary hypertension and heart disease.

The aims of this study were to identify in SSc a set of essential items for DU evaluation in clinical practice, starting from a large core of items contained in the OT1, to assess if the existing DU classification was useful and feasible in clinical practice and to investigate whether the DU categorisation was preferred to the simple classification of DU (i.e. recurrent and not recurrent).

## Methods

Observational trial on DU (OT1) is one of the five trials of the DeSScipher project (ClinicalTrials.gov; OT1 Identifier: NCT01836263, April 19, 2013).

The DeSScipher project [10] was based on the use of the EUSTAR (European Scleroderma Trials and Research group) long-term databank MEDS (Minimal Essential Data Set) accessible online [11]. The structure of the multicentre and international, prospective, longitudinal EUSTAR database has been described previously [12].

In OT1, the efficacy of different vasoactive/vasodilating drugs on DU prevention and healing was analysed considering a large number of clinical items on cutaneous lesions of the upper and lower limbs. A tailored approach of DU classification according to their main features was adopted: DU associated digital pitting scars (DPS), DU associated with calcinosis, DU due to loss of tissue not associated with DPS or calcinosis (Pure DU) (Fig. 1) and DU associated with gangrene [9]. Since recurrent DUs are a challenge in clinical practice, these data were also collected in the OT1.

For the purpose of the DeSScipher observational trials, the MEDS online database was extended and adapted according to the needs of the individual projects. The OT1-specific DeSScipher dataset included more than 30 supplementary clinical items in addition to three items on upper limb lesions contained in the original MEDS online database (digital ulcers, pitting scars on fingertips and gangrene).

OT1 data on DU were collected prospectively from April 2013 to November 2016. At the time of the analysis (November 2016), clinical data on 1749 patients enrolled into OT1 were stored in the database. Out of the items contained in the database, only those on cutaneous lesions were selected. Clinical items on upper limb DU distal to the proximal interphalangeal joints (PIP) were then identified and their completeness (completion rate) was assessed. Completeness was defined as the proportion of stored data against the potential of “100% complete” or the extent to which data were not missing [13]. Items that reached more than 60% of completeness (completion rate) were identified and inserted in a questionnaire asking which of the identified items were considered essential for DU management in everyday clinical practice. Questions about feasibility and usefulness of the DU classification were adopted in OT1 and the newly proposed DU categorisation [3]. The new DU categorisation was defined as follows:

- Episodic DU (rarely recurrent DU) defined as DU detected only at one follow-up visit and absence of DU at the remaining follow-up visits.
- Recurrent DU (frequently recurrent DU) defined as DU detected at two or more follow-up visits and absence of DU on at least one follow-up visit.
- Chronic DU defined as one or more DU and/or new DU detected at every follow-up visit.

This categorisation was published after the beginning of the OT1 study and therefore could not originally be adopted.

The questionnaire was administered to all EUSTAR centres by online survey via SurveyMonkey commercial software. The names of the EUSTAR co-workers are provided in Additional file 1.

Ethical approval of DeSScipher OT1 had been obtained from all participating centres’ local ethics committees (project coordinator’s ethics board: Ethics Review board of the Justus-Liebig University Giessen, Germany, approval no 02/13; partner centres’ ethics review boards: University of Zurich, Switzerland; University of Paris, France; University of Florence Italy; The Second University of Naples, Italy; University of Basel, Switzerland; University College of London, UK; University of Berlin Charité, Germany; University of Pécs, Hungary; University of Leeds, UK; and contributor centres’ ethics boards (additional 21 centres)). Each patient signed a written informed consent form. Moreover, there was an external data monitoring as a part of study quality control.

The assessment of the completion rate of different clinical items included in the study was performed by SPSS software, version 22. Responses to the online questionnaire were analysed by the SurveyMonkey commercial software.

## Results

OT1 contained 35 clinical items on upper and lower limb cutaneous lesions; 18 were on the upper limb DU distal to PIP (Table 1). The items on upper limb DU distal to the PIP and their data completeness are shown in italic letters in Table 1.

**Table 1:** OT1 DeSScipher items and their data completeness

OT1 DeSScipher item	Overall data completeness (%)
Pitting scars fingertips	87.3
Digital ulcers	93.4
*DU distal to the PIP*	*95.2*
*DU distal to the PIP: within last 24 weeks*	*34.6*
*DU distal to the PIP: intravenous Iloprost in last 3 months or present*	*44.7*
*DU distal to the PIP: recurrent*	*95.1*
*Upper limbs: total number of DU distal to the PIP*	*83.1*
*Upper limbs: history of DU distal to the PIP*	*91.3*
*Upper limbs: presence of infection of DU distal to the PIP*	*96.6*
*Upperlimbs: gangrene*	88.2
*Upperlimbs: previous amputation*	88.7
*Upper limbs/localisation of DU PIP: fingertip*	58.5
*Upper limbs/localisation of DU PIP: on bony prominence*	31.8
*Upper limbs/localisation of DU PIP: unknown*	14.0
*Upper limbs: number of DU defined as loss of tissue*	65.4
*Upper limbs: number of DU due to calcinosis*	66.0
*Upper limbs: number of DU due to digital pitting scars*	60.7
*Upper limbs: number DU with unknown origin*	55.3
*Upper limbs: number of new DU*	83.1
*Upper limbs: number of DU healed*	76.7
Lower limbs: total number of DU	84.7
Lower limbs: history of DU	86.2
Lower limbs: presence infection of DU	80.2
Lower limbs: gangrene	87.8
Lower limbs: previous amputation	88.2
Lower limbs/localisation of DU: patella	1.0
Lower limbs/localisation of DU: malleoli	20.8
Lower limbs/localisation of DU: calcaneus	8.3
Lower limbs/localisation of DU: toes	45.9
Lower limbs/localisation of DU: any other part of leg	14.6
Lower limbs/localisation of DU: unknown	6.2
Lower limbs: number of new DU	83.8
Lower limbs: number of DU healed	82.0
Lower limbs: peripheral arterial disease	86.8
Subcutaneous calcinosis hands	92.4

Data completeness of DeSScipher items on upper limb DU distal to PIP are in italic letters

*DU* digital ulcers, *PIP* proximal interphalangeal joints

The survey on usefulness of the items that reached ≥ 60% of completeness in the OT1 was concluded by a total of 84/148 (56.8%) EUSTAR centres. The items that obtained the highest score as essential and feasible for DU evaluation in everyday clinical practice (Table 2) were the following:

- Number of DU defined as due to loss of tissue (pure ulcers) (level of agreement 92%)
- Recurrent DU (84%)
- Number of new DU (74%)

**Table 2:** Essential clinical items for DU assessment and management

Item	Level of agreement regarding feasibility and usefulness of single items in clinical practice (%)
*Number of DU defined as loss of tissue*	*91.7*
*Recurrent DU*	*83.9*
*Number of new DU*	*73.6*
History of DU	60.9
Gangrene	60.9
Total number of DU	59.8
Infection of DU	58.6
DU distal to the proximal interphalangeal joints	50.6
Previous amputation	49.4
Number of DU due to calcinosis	46.4
Number of DU due to DPS	45.2
Number of healed DU	24.1

The items that reached level of agreement ≥ 70% are in italic letters

*DU* digital ulcers, *DPS* digital pitting scars

A significant number of centres (64%) agreed that the DU classification adopted in OT1 [9] was useful to identify DU and their characteristics fundamental to shape the management in everyday clinical practice. Concerning the new categorisation of DU [3], 80% of the centres preferred the distinction in episodic, recurrent and chronic DU compared to the simple division in recurrent and not recurrent DU.

## Discussion

This study introduces for the first time the concept of essential clinical items for the evaluation and management of DU in SSc. These essential items might become a useful tool for physicians treating DU in everyday clinical practice and may also become outcome measures to be used in clinical trials.

The item considered as the most important for DU management was the number of DU defined as a loss of tissue, voted by more than 90% of participants. Thus, the DU due to loss of tissue or pure DU, referring to a DU occurring neither in association with DPS nor with calcinosis, was considered as the most important form of DU in SSc. This finding underlines the perceived importance of the clinical burden of this type of DU, since they usually represent the most severe type of DU where vasoactive/vasodilating drugs used for DU treatment have been tested. It is interesting to remark that the assessment of other types of DU, as those due to DPS or to calcinosis, was not evaluated as important in clinical practice. This may likely reflect the fact that these lesions are usually considered mild and not disabling.

Recurrent DU and number of new DU were the second and the third chosen essential items, respectively.

The number of new DU was considered more important than the number of healed DU. In fact, the DU occurrence has been correlated to a worse outcome and a poor quality of life in large prospective SSc cohorts [4, 5, 14, 15]. The number of new DU was included by participants among the essential items, being probably considered as an indicator of clinical worsening and more severe disease.

Unexpectedly, only 20% of centres considered the number of healed DU useful and feasible for DU management in clinical practice as this was the least voted item. This result may reflect a difficulty in assessing the healing of each DU in clinical practice, since not always all patients are seen at time interval useful to depict the healing of all lesions.

Interestingly, only half of the participants chose DU distal to the proximal interphalangeal joints as an important item. This indicates that not all clinicians considered the site of DU important for their management. However, DUs located on the fingertips usually follow tissue damage due to chronic ischaemia [14], while DUs on other locations may also be due to cutaneous retraction and microtrauma, thus being less responsive to vasodilating/vasoactive drugs.

Surprisingly, for less than 60% of the participants, the presence of infection was essential for DU management. This may indicate insufficient attention to this item even by centres expert in SSc management. Accordingly, there is a scarce number of scientific publications on infectious complications of DU in SSc [3, 16, 17], and up to now, no study has addressed the impact of infection on DU healing. Data published till now indicate that infection is frequent in patients with DU. Giuggioli et al. observed in a retrospective study that 51% of 82 SSc patients with DU presented infected DU over a three-year observational period [17]. Moreover, in the analysis of 1459 patients taking part of the large DUO registry, soft tissue infection requiring systemic antibiotics has been observed in 60% of patients with one or more DU at every follow-up visit over 2 years [3]. In addition, it is a common clinical observation that infected DUs have impaired healing potential in SSc, and it has been shown that infected wounds and ulcers have a worse outcome in other clinical settings [18].

It is of note that gangrene and previous amputation were considered important for DU management only by 60% and 50% of centres, respectively. However, a recent study on more than 4600 patients demonstrated that gangrene is still a common event in current practice, occurring in 18% of patients with SSc-related DU [19].

Recurrent DUs are a real clinical challenge in SSc. Accordingly, recurrent DU represented the second most voted clinical item in our study. Simple distinction in recurrent and not recurrent DU may not fully depict the level of DU-related disease burden. For this reason, a new categorisation, based on the longitudinal pattern of DU recurrence over the 2 years in the registry containing more than 1400 patients across Europe has been recently proposed [3]. The participating centres in our study have recognised the importance of this categorisation, as more than 80% of them agreed on its utility and feasibility in clinical practice. In fact, it may help to identify patients with more severe DU disease burden that may require more intensive treatment, as already suggested [3].

The aim of the OT1 was to evaluate the best vasodilating/vasoactive therapy for DU prevention and healing through observational non-interventional design. In order to distinguish between different types of DU that might have different response to the treatment, OT1 needed to classify DU. Since there is no universally accepted classification of DU in SSc, OT1 adopted the one proposed by Amanzi et al. [9] based on observations extrapolated from real life data on more than 1500 DU [9]. Our study has shown that this classification may be useful and feasible in everyday clinical practice as indicated by 64% of the participating centres.

The strength of this study is that the items were already pre-selected based on the analysis of data availability in the DeSScipher project, the first prospective multi-centric European study that expressly addressed DU management in SSc, with a study population of more than 1600 SSc patients. More than 80 expert centres in SSc management across the world were included in this survey. The limitation of this project is that the online survey was based on the opinion of a single expert of each individual centre. In addition, the survey contained only clinical items (DU features assessed by the clinical history and/or simple clinical examination). Several laboratory and instrumental items have been collected in the OT1 database. The importance of some of these parameters for DU management, such as capillaroscopic findings shown to be risk factors for DU occurrence [20, 21], should be addressed in the future.

Our findings now need prospective validation using data-driven approach on large SSc cohorts in order to confirm the real usefulness of these essential items and the role of the proposed DU classification and categorisation in real-life clinical practice.

## Conclusions

For clinical practice, DeSScipher/EUSTAR centres identified only three essential items for DU evaluation. They considered the proposed classification and categorisation of DU as useful and feasible. The set of items needs to be further validated while further implementation of DU classification and categorisation is warranted.

## Additional file

Additional file 1: EUSTAR co-workers. Full list of EUSTAR co-workers according the numerical order of centres. (DOCX 26 kb)

## Abbreviations

DPS: Digital pitting scars
DU: Digital ulcers
EUSTAR: European Scleroderma Trials and Research group
MEDS: Minimal Essential Data Set
OTs: Observational trials
PIP: Proximal interphalangeal joints
SSc: Systemic sclerosis
