# Supplementary material for: Classification, categorization and essential items for digital ulcer evaluation in systemic sclerosis: a DeSScipher/European Scleroderma Trials and Research group (EUSTAR) survey
Source: Arthritis Res Ther. 2019 Jan 24;21:35. doi: 10.1186/s13075-019-1822-1 (PMC6346551; doi:10.1186/s13075-019-1822-1)
Supplement: Supplementary file 1 — EUSTAR co-workers. Full list of EUSTAR co-workers according the numerical order of centres. (DOCX 26 kb) [file 13075_2019_1822_MOESM1_ESM.docx]

**EUSTAR co-workers** (according the numerical order of centres):

Cosimo Bruni (Department of Experimental and Clinical Medicine, University of Florence, Division of Rheumatology AOUC, Florence Italy); Giovanni Lapadula, Florenzo Iannone, Fabio Cacciapaglia (Rheumatology Unit-DiMIMP, School of Medicine University of Bari, Italy); Suzana Jordan, Mike Becker, Carina Mihai, Rucsandra Dobrota (Department of Rheumatology, University Hospital Zurich, Zurich, Switzerland); Radim Becvarar, Radim Becvar, Michal Tomcík (Institute of Rheumatology, 1st Medical School, Charles University, Prague, Czech Republic); Stanislaw Sierakowsky, Otylia Kowal Bielecka (Department of Rheumatology and Internal Medicine, Medical University of Bialystok, Bialystok, Poland);Maurizio Cutolo, Alberto Sulli, Barbara Ruaro, Elisa Alessandri, Carmen Pizzorni, Sabrina Paolino (Research Laboratory and Division of Rheumatology, Department of Internal Medicine, University of Genova, Italy); Gabriele Valentini, Antonella Riccardi, Veronica Giacco, Valentina Messiniti, Rosaria Irace (Department of Clinical and Experimental Medicine ‘F-Magrassi’ II Policlinico, Unit of Rheumatology, Naples, Italy); Elise Siegert, Claudia Kedor, Vincent Casteleyn, Christine March, Jakob Hoeppner (Department of Rheumatology, Charitè University Hospital, Berlin); Simona Rednic, Ana Petcu, Iulia Szabo (Department of Rheumatology, University of Medicine and Pharmacy ‘Iuliu Hatieganu’ Cluj, Cluj-Napoca, Romania); Muriel Elhai (Department of Rheumatology, University Paris Descartes and Cochin Hospital, Paris, France); P. Vlachoyiannopoulos (Department of Pathopysiology, Medical School, National University of Athens, Greece); Carlomaurizio Montecucco, Roberto Caporali, Veronica Codullo (Unita’ Operativa e Cattedra di Reumatologia, IRCCS Policlinico S Matteo, Pavia, Italy); Jiri Stork (Charles University in Prague, First Faculty of Medicine and General University Hospital, Prague, Czech Republic); Murat Inanc (Istanbul Medical Faculty, Department of Internal Medicine, Division of Rheumatology, Capa, Istanbul, Turkey); Patricia E. Carreira, Beatriz E Joven (Division of Rheumatology, Hospital 12 de Octubre, Madrid, Spain); Srdan Novak, Felina Anic (Department of Rheumatology and Clinical Immunology, Internal Medicine, KBC Rijeka, Croatia); Cecilia Varju, Tünde Minier (Department of Immunology and Rheumatology, Faculty of Medicine, University of Pécs, Hungary); Carlo Chizzolini, Danièle Allali (Department of Immunology and Allergy, University Hospital, Geneva, Switzerland); Eugene J. Kucharz, Magdalena Kopec-Medrek, Anida Grosicka, Malgorzata Widuchowska (Department of Internal Medicine and Rheumatology, Medical University of Silesia, Katowice, Poland); Andrea Doria, Elisabetta Zanatta (Rheumatology Unit, Department of Medicine, University of Padova, Italy); Alenka Sipek Dolnicar (University Medical Center Ljublijana, Division of Internal Medicine, Department of Rheumatology, Ljubliana, Slovenia); Bernard Coleiro (‘Stella Maris’, Balzan, Malta); Armando Gabrielli, Lucia Manfredi, Alessia Ferrarini (Dipartimento di Scienze Cliniche e Molecolari, Clinica Medica, Università Politecnica delle Marche, Ancona, Italy); Dominique Farge Bancel, Adrian Hij, Pauline Lansiaux (Department of Internal Medicine, Hospital Saint-Louis, Paris, France); Paolo Airò, Maria-Grazia Lazzaroni (Spedali Civili di Brescia, Servizio di Reumatologia Allergologia e Immunologia Clinica, Brescia, Italy); Roger Hesselstrand, Dirk Wuttge, Kristofer Andréasson (Department of Rheumatology, Lund University, Lund, Sweden); Duska Martinovic, Ivona Bozic ; Mislav Radic (Department of Internal Medicine, Clinical Hospital of Split, Croatia); Alexandra Balbir-Gurman, Yolanda Braun-Moscovici (B. Shine Rheumatology Unit, Rambam Health Care Campus, Rappaport Faculty of Medicine, Technion, Haifa, Israel); Andrea Lo Monaco, Federica Furini (Department of Clinical and Experimental Medicine, Rheumatology Unit, University of Ferrara, Italy); Nicolas Hunzelmann, Pia Moinzadeh, Thomas Krieg (Department of Dermatology, University Hospital Cologne, Germany); Raffaele Pellerito (Ospedale Mauriziano, Centro di Reumatologia, Torino, Italy); Cristian Caimmi, Bertoldo Eugenia (Rheumatology Unit, University of Verona, Verona, Italy); Jadranka Morovic-Vergles, Ivana Melanie Culo (Pročelnica Zavoda za kl imunologiju, alergologiju i reumatologiju, Klinike za unutarnje bolesti, Medicinskog fakulteta Sveučilišta u Zagrebu, Zagreb, Croatia); Nemanja Damjanov (Institute of Rheumatology, Belgrade, Serbia); Vera Ortiz Santamaria (Rheumatology Granollers General Hospital, Barcelona, Spain); Stefan Heitmann, Madeleine Codagnone, Johannes Pflugfelder (Department of Rheumatology, Marienhospital Stuttgart, Germany); Dorota Krasowska, Malgorzata Michalska-Jakubus (Department of Dermatology, Medical University of Lublin, Poland); Matthias Seidel (Medizinische Universitäts-Poliklinik, Department of Rheumatology, Bonn, Germany); Paul Hasler, Samuel Kretzschmar (Kantonsspital Aarau, Rheumaklinik und Institut für Physikalische Medizin und Rehabilitation Kantonsspital Aarau, Switzerland); Michaela Köhm (Klinikum der Johann Wolfgang Goethe Universität, Medizinische Klinik III, Rheumatologische Ambulanz, Frankfurt am Main, Germany); Ivan Foeldvari, Nicola Helmus (Hamburger Zentrum für Kinder- und Jugendrheumatologie Kompetenz-Zentrum für Uveiits und Sklerodermie im Kindes- und Jugendalter An der Schön Klinik Hamburg Eilbek, Hamburg, Germany); Gianluigi Bajocchi (Arcispedale Santa Maria Nuova, Dipartimento Area Medica I, U.O. di Reumatologia, Reggio Emilia, Italy); Maria Joao Salvador, José Antonio Pereira Da Silva (Rheumatology Department, Hospitais da Universidade, Coimbra, Portugal); Bojana Stamenkovic, Aleksandra Stankovic (Institute for Prevention, Treatment and Rehabilitation of Rheumatic and Cardiovascular Diseases, Niska Banja, Serbia); Carlo Francesco Selmi, Maria De Santis, Angela Ceribelli (Division of Rheumatology and Clinical Immunology Humanitas Clinical and Research Center BIOMETRA Department, University of Milan, Italy); Mohammed Tikly (Rheumatology Unit, Department of Medicine Chris Hani Haragwanath, Hospital and University of the Witwatersrand, Johannesburg, South Africa); Lidia P. Ananieva, Ludmila Garzanova, Olga Koneva, Maya Starovoytova (Institute of Rheumatology, Russian Academy of Medical Science, Moscow, Russia); Ariane Herrick (Hope/Hospital University of Manchester Rheumatic Diseases Centre, Salford, United Kingdom); Raffaella Scorza (U.O. Immunologia Clinica, Centro di Riferimento per le Malattie Autoimmuni Sistemiche, Milano, Italy); Francesco Puppo (Clinica di Medicina Interna ad orientamento immunologico-Università di Genova, IRCCS Azienda Ospedaliero-Universitaria,Università San Martino, Genova, Italy); Merete Engelhart (Department of Rheumatology, University Hospital of Gentofte, Hellerup, Denmark); Gabriela Szücs, Szilvia Szamosi (Third Department of Medicine, Rheumatology Division; University of Debrecen, Hungary); Carlos de la Puente, Cristina Sobrino Grande, María Jesus García Villanueva (Servicio de Reumatología, Hospital Ramon Y Cajal, Madrid, Spain); Anna-Maria Hoffmann-Vold, Øyvind Midtvedt (Department of Rheumatology, Rikshospitalet University Hospital, Oslo, Norway); Eric Hachulla, David Launay, Vincent Sobanski (Department of Internal Medicine, Hôpital Claude Huriez, Lille, France); Valeria Riccieri, Massimiliano Vasile, Katia Stefantoni (Department of Internal Medicine and Medical Specialities, ‘Sapienza’ University of Rome, Italy);Ruxandra Maria Ionescu, Daniela Opris, Laura Groseanu (Department of Rheumatology, St. Mary Hospital, Carol Davila, University of Medicine and Pharmacy, Bucharest, Romania); Ami A. Shah, Adrianne Woods (Johns Hopkins University Division of Rheumatology, Johns Hopkins School of Medicine, Baltimore, USA); Carina Mihai, Ana Maria Gheorghiu, Mihai Bojinca (Department of Internal Medicine and Rheumatology, Cantacuzino Hospital, Carol Davila University of Medicine and Pharmacy, Bucharest, Romania); Cord Sunderkötter, Jan Ehrchen (Department of Dermatology, University of Münster, Germany); Jörg HW Distler (Department of Internal Medicine 3, University Hospital Erlangen, Germany); Francesca Ingegnoli (Division of Rheumatology, Istituto Gaetano Pini, Department of Clinical Sciences and Community Health, University of Milano, Milano, Italy); Luc Mouthon, Bertrand Dunogue, Benjamin Chaigne, Paul Legendre (Department of Internal Medicine, Hôpital Cochin, Paris, France); Vanessa Smith (University of Ghent, Department of Rheumatology, Gent, Belgium); Francesco P. Cantatore, Ada Corrado (U.O. Reumatologia-Università degli Studi di Foggia, Ospedale ‘Col. D'Avanzo’, Foggia, Italy); Susanne Ullman (University Hospital of Copenhagen, Denmark); Carlos A. von Muhlen (Rheuma Clinic, Porto Alegre, Brazil); Maria Rosa Pozzi (Dipartimento di Medicina, Ospedale San Gerardo, Monza, Italy); Kilian Eyerich, Felix Lauffer (Department of Dermatology and Allergy of the TU Munich, Germany); Piotr Wiland, Magdalena Szmyrka-Kaczmarek, Renata Sokolik, Ewa Morgiel, Marta Madej (Department of Rheumatology and Internal Diseases, Wroclaw University of Medicine, Wroclaw, Poland); Marie Vanthuyne, Frederic Houssiau (Université Catholique de Louvain, Cliniques Universitaires St-Luc, Bruxelles, Belgium; Juan Jose Alegre-Sancho (Hospital Universitario Dr Peset, Valencia, Spain); Kristine Herrmann, Claudia Guenther (Division of Rheumatology Department of Medicine III, and Department of Dermatology, University Medical Center Carl Gustav Carus Technical University of Dresden, Germany); Ellen De Langhe, Rene Westhovens, Jan Lenaerts (Catholic University of Leuven, Department of Rheumatology, Leuven, Belgium); Branimir Anic, Marko Baresic, Miroslav Mayer (University Hospital Centre Zagreb, Division of Clinical Immunology and Rheumatology, Department of Medicine, Zagreb, Croatia); Maria Üprus, Kati Otsa (East-Tallin Central Hospital, Department of Rheumatology, Tallin, Estonia); Sule Yavuz (University of Marmara, Dept. of Rheumatology, Altunizade, Istanbul, Turkey); Brigitte Granel (Service de Médecine Interne, Hôpital Nord de Marseille, Chemin des Bourrelys, Marseille, France); Sebastião Cezar Radominski, Carolina de Souza Müller, Valderílio Feijó (Azevedo Hospital de Clínicas da Universidade Federal do Paraná, Curitiba - Paraná, Brasil); Fabian Mendoza, Joanna Busquets (Thomas Jefferson Scleroderma Center, Division of Rheumatology and Jefferson Institute of Molecular Medicine, Philadelphia, USA); Svetlana Agachi, Sergei Popa (Municipal Centres of Research in Scleroderma, Hospital ‘Sacred Trinity’, Department of Rheumatology/Department of Rheumatology, Republican Clinical Hospital, Chisinau, Republic of Moldova); Thierry Zenone (Department of Medicine, Unit of Internal Medicine, Valence cedex 9, France); Margarita Pileckyte; Simon Stebbings, Sarah Jordan (Dunedin School of Medicine, Dunedin, New Zealand); Alessandro Mathieu, Alessandra Vacca (II Chair of Rheumatology, University of Cagliari-Policlinico Universitario, Monserrato (CA), Italy); Percival Degrava Sampaio Barros (University of São Paulo-Rheumatology Division, Faculdade de Medicina de Universidade de São Paulo, Brasil); Lisa Stamp (Department of Medicine, University of Otago Christchurch, New Zealand); Kamal Solanki, Cherumi Silva, Joanne Schollum, Helen Barns-Graham Waikato (University Hospital, Rheumatology Unit, Hamilton City, New Zealand); Douglas Veale (Department of Rheumatology, Bone and Joint Unit, St. Vincent's University Hospital, Dublin, Ireland); Esthela Loyo, Carmen Tineo, Glenny Paulino (Reumatologia e Inmunologia Clinica, Hospital Regional Universitario Jose Ma Cabral y Baez, Clinica Corominas, Santiago, Dominican Republic); Mengtao Li (Department of Rheumatology, Peking Union Medical College Hospital (West Campus), Chinese Academy of Medical Sciences, Beijing, China); Walid Ahmed Abdel Atty Mohamed (Alexandria University, Unit of Rheumatology,Alexandria Egypt); Edoardo Rosato, Antonio Amoroso, Antonietta Gigante (Centro per la Sclerosi Sistemica - Dipartimento di Medicina Clinica, Università La Sapienza, Policlinico Umberto I, Roma, Italy); Fahrettin Oksel, Figen Yargucu, (Ege University, Faculty of Medicine, Dept. of Internal Medicine, Division of Rheumatology, Bornova, Izmir, Turkey); Cristina-Mihaela Tanaseanu, Monica Popescu, Alina Dumitrascu, Isabela Tiglea (Clinical Emergency Hospital St. Pantelimon, Bucharest, Romania); Rosario Foti, Alessia Benenati, Elisa Visalli (U.O. di Reumatologia, A.O.U. Policlinico Vittorio Emanuele, Catania, Italy); Codrina Ancuta (Division of Rheumatology and Rehabilitation GR.T.Popa, Center for Biomedical Research, European Center for Translational Research, "GR.T.Popa" University of Medicine and Pharmacy, Rehabilitation Hospital, Iasi, Romania); Peter Villiger, Johannes Fröhlich, Diana Dan, Sabine Adler (Department of Rheumatology and Clinical Immunology/Allergology, Inselspital, University of Bern, Switzerland); Jacob van Laar, Kamran Naraghi (James Cook University Hospital, Middlesbrough, United Kingdom); Cristiane Kayser, Andrade Luis Eduardo C (Universidade Federal de São Paulo, Disciplina de Reumatologia, São Paulo, Brasil); Nihal Fathi, Safa Alii, Marrow Ahmed, Samar Hasaneen Eman El Hakeem (Assiut and Sohage University Hospital, Rheumatology Department, Assiut University Hospital, Egypt); Paloma García de la Peña Lefebvre, Jorge Juan González Martín (Hospital Universitario Madrid Norte Sanchinarro, Madrid, Spain); Jean Sibilia, Emmanuel Chatelus, Jacques Eric Gottenberg, Hélène Chifflot (University Hospital of Strasbourg, Department of Rheumatology, Hôpital de Hautepierre, Service de Rhumatologie, Strasbourg Cedex, France); Irena Litinsky (Department of Rheumatology, Tel Aviv Sourasky Medical Center, Tel Aviv, Israel); Sookhoe Eng, Gianluca Bagnato (Scleroderma Programme, Institute of Molecular Medicine, Division of Musculoskeletal Diseases, University of Leeds, United Kingdom); Goda Seskute, Irena Butrimiene, Rita Rugiene, Diana Karpec (State Research Institute for Innovative Medicine, Vilnius University, Vilnius, Lithuania); Lesley Ann Saketkoo, Melanie Pascal (Tulane University Lung Center, Tulane/University Medical Center Scleroderma and Sarcoidosis Patient Care and Research Center, New Orleans, USA); Eduardo Kerzberg (Rheumatology Department, Ramos Mejía Hospital, Buenos Aires, Argentina); Washington Bianchi, Sueli Carneiro, Giselle Baptista Maretti, Dante Valdetaro Bianchi (Department of Rheumatology-Santa Casa da Misericórdia do Rio de Janeiro, Rio de Janeiro, Brasil); Ivan Castellví, Milena Millan (Hospital de la Santa Creu i Sant Pau, Barcelona, Spain); Massimiliano Limonta (USSD Reumatologia, Ospedali Riuniti di Bergamo, Italy); Doron Rimar, Gleb Slobodin, Itzhak Rosner (Rheumatology, Bnai Zion Medical Center/Technion, Haifa, Israel); Maura Couto (Unidade de Reumatologia de Viseu, Centro Hospitalar Tondela-Viseu (Unidade de Reumatologia), Viseu-Portugal); François Spertini, Camillo Ribi, Guillaume Buss (Department of Rheumatology, Clinical Immunology and Allergy, Lausanne, Switzerland); Antonella Marcoccia, Francesco Bondanini, Aldo Ciani (Capillaroscopic Unit - Sandro Pertini Hospital, Roma, Italy); Sarah Kahl (Universitätsklinikum Schleswig-Holstein, Campus Lübeck Innere Medizin/Rheumatologie/Immunologie Rheumaklinik Bad Bramstedt, Germany); Vivien M. Hsu (Rutgers- RWJ Scleroderma Program, Program Director, Rutgers-RWJ Rheumatology Fellowship Program, New Brunswick, USA); Thierry Martin, Vincent Poindron, Kilifa Meghit (Clinical Immunology Internal Medicine. National Referral Center for Systemic Autoimmune Diseases, Nouvel Hopital Civil, Strasbourg, France); Sergey Moiseev, Pavel Novikov (Clinic of Nephrology, Internal and Occupational Diseases, Rossolimo, Moscow, Russia); Lorinda S Chung, Kathleen Kolstad, Marianna Stark (Department of Dermatology Stanford University School of Medicine, Redwood City, USA); Tim Schmeiser, Astrid Thiele (Krankenhaus St. Josef, Wuppertal-Elberfeld, Germany); Dominik Majewski (Department of Rheumatology and Internal Medicine Poznan University, Poznań, Poland); Julia Martínez-Barrio, Javier López Longo (Department of Rheumatology, Gregorio Marañón Univeristy Hospital, Madrid, Spain); Vera Bernardino, Maria Francisca Moraes-Fontes, Ana Catarina Rodrigues (Unidade de Doencas Autoimunes, Hospital Curry Cabral, Centro Hospitalar Lisboa Central, Lisboa, Portugal); Sabine Sommerlatte, Sebastian Jendreck, Sabrina Arnold (Universitätsklinik Lübeck, Germany); Lèlita Santos (Consulta de Doenças Autoimunes Sistémicas Centro Hospitalar e Universitário de Coimbra – CHUC, EPE, Coimbra, Portugal); Yair Levy (Internal medicine, Meir Medical Center, Kfar Saba, Israel); Elena Rezuș, Anca Cardoneanu, Alexandra Burlui (Rheumatology Department, "Grigore T.Popa" University of Medicine and Pharmacy Iasi, 1st Rheumatology Clinic, Clinical Rehabilitation Hospital Iasi); Omer Nuri Pamuk (Trakya University Medical Faculty, Department of Internal Medicine, Division of Rheumatology, Edirne, Turkey); Daniel Brito de Araujo (Universidade Federal de Pelotas, Department: Internal Medicine, Pelotas, Brazil); Piercarlo Sarzi Putiini, Rossella Talotta, Sara Bongiovanni (University Hospital Luigi Sacco, Milan, Italy); Hadi Poormoghim, Simin Almasi, Elham Andalib (Scleroderma Study group, Department of Rheumatology. Firoozgar hospital, Tehran, Iran); Ina Kötter, Martin Krusche (Rheumatologie, Klinische Immunologie, Nephrologie Asklepios Klinik Altona Hamburg, Germany); Giovanna Cuomo, Fiammetta Danzo, Francesco Masini (UOC Medicina Interna, Università della Campania, Napoli, Italy); Francis Gaches, Florian Catros, Martin Michaud (Centre de Compétence Maladies Lysosomales, Hôpital Joseph Ducuing, Toulouse, France); Laura Belloli (Struttura Complessa di Reumatologia, Dipartimento Medico Polispecialistico ASST Grande Ospedale Metropolitano Niguarda, Milan, Italy); Petros Sfikakis, Maria, Tektonidou (Rheumatology Unit, First Propaedeutic and Internal Medicine, Athens University Medical School. Athens, Greece); Juliana Markus (Serviço de Reumatologia, Hospital de Clínicas da Universidade Federal de Uberlândia, Uberlândia, Brasil); Daniel Furst, Philip Clements, Suzanne Kafaja (Arthritis Association of Southern California, Los Angeles, USA); Adriana Apostol, Ana-Maria Ramazan (Rheumatology Department, Spitalul Clinic Judetean de Urgenta, “Sf Apostol Andrei”, Constanta City, Romania); J.K. de Vries-Bouwstra, H.U. Scherer (Department of Rheumatology, Leiden University Medical Center, Leiden, The Netherlands); Marie Elise Truchetet (CHU de Bordeaux Rheumatology department; Bordeaux, France); Patrick Jego, Alain Lescoat (Centre Hospitalier Universitaire De Rennes, Rennes, France).
